# Supplementary material for: Analysis of PPARγ Signaling Activity in Psoriasis
Source: Int J Mol Sci. 2021 Aug 10;22(16):8603. doi: 10.3390/ijms22168603 (PMC8395241; doi:10.3390/ijms22168603)
Supplement: Supplementary file 1 [file ijms-22-08603-s001.zip › Supplemental materials_Analysis of PPARg signaling activity in psoriasis/Pathway models/Models images and html files/PPARG inhibits phenotypes assotiated with psoriasis/189901.html]

anagen


# Cell Process anagen

|  |  |
| --- | --- |
| URN | urn:agi-gocellproc:0042640 |
| Total Entities | 0 |
| Connectivity | 419 |
| Name | anagen |

---

|  |  |
| --- | --- |
| Pathway | Hair Follicle Keratinocyte Apoptosis |
|  | Telogen Maintenance in Androgenic Alopecia |
|  | Androgens Promote Scalp Dermal Papilla Regression |
|  | PPARG inhibits phenotypes assotiated with psoriasis |
|  | efects selected |

---

|  |  |
| --- | --- |
| MedScan ID | 4142640 |

---

|  |  |
| --- | --- |
| Alias | anagen |
|  | hair growth cycles |
|  | anagens |
|  | hair growth cycle |
|  | cycles of hair growth |
|  | cycle of hair growth |
|  | growth cycle of the hair |
|  | hair growth cycling |

---

|  |  |
| --- | --- |
| GO ID | 0042640 |

---
